# Comprehensive clinical evaluation of deep learning-based auto-segmentation for radiotherapy in patients with cervical cancer

**DOI:** 10.3389/fonc.2023.1119008

**Published:** 2023-04-28

**Authors:** Seung Yeun Chung, Jee Suk Chang, Yong Bae Kim

**Affiliations:** ^1^ Department of Radiation Oncology, Yonsei University College of Medicine, Seoul, Republic of Korea; ^2^ Department of Radiation Oncology, Ajou University School of Medicine, Suwon, Republic of Korea

**Keywords:** cervical cancer, radiotherapy, auto-segmentation, deep learning, auto-contouring

## Abstract

**Background and purpose:**

Deep learning-based models have been actively investigated for various aspects of radiotherapy. However, for cervical cancer, only a few studies dealing with the auto-segmentation of organs-at-risk (OARs) and clinical target volumes (CTVs) exist. This study aimed to train a deep learning-based auto-segmentation model for OAR/CTVs for patients with cervical cancer undergoing radiotherapy and to evaluate the model’s feasibility and efficacy with not only geometric indices but also comprehensive clinical evaluation.

**Materials and methods:**

A total of 180 abdominopelvic computed tomography images were included (training set, 165; validation set, 15). Geometric indices such as the Dice similarity coefficient (DSC) and the 95% Hausdorff distance (HD) were analyzed. A Turing test was performed and physicians from other institutions were asked to delineate contours with and without using auto-segmented contours to assess inter-physician heterogeneity and contouring time.

**Results:**

The correlation between the manual and auto-segmented contours was acceptable for the anorectum, bladder, spinal cord, cauda equina, right and left femoral heads, bowel bag, uterocervix, liver, and left and right kidneys (DSC greater than 0.80). The stomach and duodenum showed DSCs of 0.67 and 0.73, respectively. CTVs showed DSCs between 0.75 and 0.80. Turing test results were favorable for most OARs and CTVs. No auto-segmented contours had large, obvious errors. The median overall satisfaction score of the participating physicians was 7 out of 10. Auto-segmentation reduced heterogeneity and shortened contouring time by 30 min among radiation oncologists from different institutions. Most participants favored the auto-contouring system.

**Conclusion:**

The proposed deep learning-based auto-segmentation model may be an efficient tool for patients with cervical cancer undergoing radiotherapy. Although the current model may not completely replace humans, it can serve as a useful and efficient tool in real-world clinics.

## 1 Introduction

Radiotherapy (RT) plays a crucial role in the curative treatment of locally advanced and early stage cervical cancer (1). Definitive RT generally includes the pelvic area and, in some cases, nodal area up to the para-aortic lymph nodes (2, 3). Many institutions use intensity-modulated RT (IMRT) for cervical cancer, which allows the delivery of a curative dose of radiation to the target while minimizing toxicities to nearby organs-at-risk (OAR). The benefit of IMRT in patients with cervical cancer has been demonstrated in a randomized controlled trial, with reduced toxicity but no difference in disease outcomes (4). However, compared to RT using conventional techniques, the planning process for IMRT is far more complicated.

For RT, a computed tomography (CT) simulation is necessary for treatment planning. Radiation oncologists contour both target volumes and OAR for every CT slice. Target volumes consist of gross tumor volume and clinical target volume (CTV), including microscopic disease and nodal areas. In cervical cancer, the anorectum, sigmoid colon, bowel, bladder, uterocervix, spinal cord, cauda equina, stomach, duodenum, liver, right and left kidneys, and femoral head can be included as OARs for treatment planning. All these contours need to be precisely contoured in every CT slice for accurate treatment planning.

Consequently, the workload of radiation oncologists has increased considerably. Consequently, increased workloads lead to increased time for preparing IMRT, which may also lead to a delay in the start of treatment for patients. Furthermore, with multiple OAR and target volumes, there may be increased heterogeneity in the definition of contours. Large heterogeneity among radiation oncologists has been shown to affect the quality of treatment (5, 6).

Deep learning has attracted substantial interest in various fields of medicine (7–9). In the field of radiation oncology, deep learning-based models have been actively investigated for various aspects of RT (10). Auto-segmentation tools have been introduced in head and neck and prostate cancer (11, 12). For cervical cancer, most research concerning auto-segmentation has focused on gross tumor segmentation (13, 14) with few studies on the auto-segmentation of OAR/CTVs on a simulated CT scan (15).

In this study, we attempted to train a deep learning-based auto-segmentation model for OAR and CTV in patients with cervical cancer undergoing RT and to evaluate the feasibility and efficacy of the model.

## 2 Materials and methods

This study was approved by the Institutional Review Board of Severance Hospital (IRB: 4-2021-0605). Informed consent was not required due to the retrospective nature of this study. This study included the planning CT images of 182 patients who underwent definitive RT as the first treatment modality for pathologically diagnosed cervical cancer between January 2016 and May 2020. Out of the 182 patients, 16 (9%) were diagnosed with stage I cervical cancer, 54 (30%) with stage II, 96 (53%) with stage III, and 16 (9%) with stage IV. The CT scans of patients who underwent surgery prior to RT were excluded. Contrast-enhanced planning CT scans (Somatom Sensation Open syngo CT 2009E, Siemens and Aquilion TSX-201A, Toshiba) were performed 1 min after the administration of 80–90 mL of intravenous contrast (iohexol, 84.11 g/130 mL, depending on the patient’s weight). Planning CT scans were performed approximately 1 week prior to RT, with a slice thickness of 3 mm. The setup for all planning CT scans was the supine position.

For homogeneity, a single expert radiation oncologist, blinded to patient information, delineated CTVs and OARs within 3 months. The initial contours used for the actual treatment were not used in this study. The OARs were contoured according to the Radiation Therapy Oncology Group guidelines (16). The rectum, bowel bag, bladder, uterocervix, spinal cord, cauda equina, stomach, duodenum, liver, right kidney, left kidney, right femoral head, and left femoral head were contoured as the OARs. For the target volume, various recommendations and guidelines were reviewed, and the pictorial atlas derived by consensus best practice delineation was used (2, 3, 17–20). CTV1 included the uterus, ovaries (if visible), gross tumor, cervix, bilateral parametria, and vagina. CTV2 included pelvic nodal groups, such as the common iliac, external iliac, internal iliac, obturator, and presacral lymph node. CTV3 included the para-aortic nodal chain.

**Figure 1:**
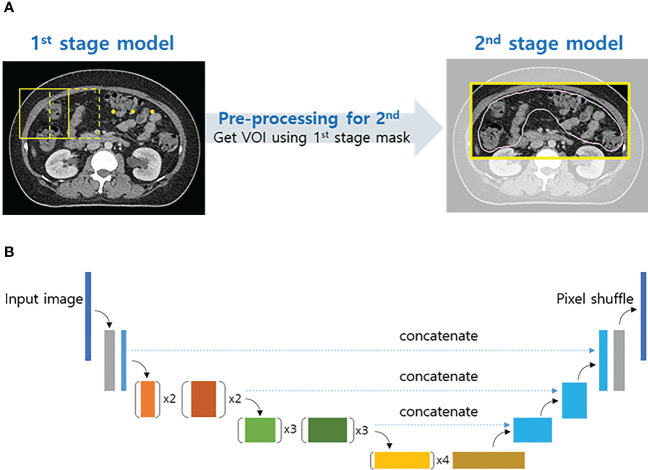
Two-stage structure algorithm for auto-segmentation **(A)** and schematic of the proposed convolutional neural network architecture (U-Net with EfficientNet-B0) **(B)**.

Among the 182 planning CT images, two were excluded because of serious metal artifacts and prone positioning. Finally, 180 female abdominopelvic CT images were included, of which 165 were used as the training set and 15 as the validation set. The algorithm for auto-segmentation was designed as a two-stage structure to improve segmentation performance (**Figure 1A**), and a U-Net structure using EfficientNet-B0 as the backbone was used as the network model (**Figure 1B**). The details of the algorithm are explained in the **Supplementary Material**.

The Dice similarity coefficient (DSC) and 95% Hausdorff distance (HD), which are the most commonly used geometrical indices, were used to compare the two different sets of contours. DSC is a measure of the overlap between two sets of contours, ranging from “0” to “1,” in which “1” implies a complete overlap. HD is the maximum distance of a set contour to the nearest point of the contour of another set. Therefore, 0 mm indicates complete overlap for HD.

As a method for subjective evaluation, the Turing test, also known as the imitation game, was used to assess whether the auto-segmented contours were distinguishable from the manual contours (21). The CT slices from the validation cases were randomly selected for each OAR/CTV. A survey, including the Turing test, was conducted using a web interface (**Supplementary Figure 1**) and consisted of four sections.

*Section I. Single contour: “How was this contour drawn?”*

Answer: By human or by computer

*Section II. Two contour sets: “Which contour set do you prefer?”*

Answer: Set A or B

*Section III. Alternative questions: “You have been asked to review these contours for clinical use by a colleague. Would you:”*

Answer:

Require them to be corrected; there are large, obvious errors.

Require them to be corrected; there are minor errors.

Accept them as they are; there are minor errors.

Accept them as they are; the contours are very precise.

*Section IV. Overall satisfaction score: “How much are you satisfied with auto-segmentation for actual clinical use?”*

Answer: 0 (worst) to 10 (best)

Sections I to III consisted of 80 questions each. Section IV, assessing physician satisfaction, consisted of five questions, one for each case.

To observe a reduction in inter-physician heterogeneity and time, 10 radiation oncologists, experts in gynecologic cancer, from six different institutions were asked to delineate OAR/CTVs on an anonymized CT image initially and record the time to complete the process. They were then asked to delineate OAR/CTVs using auto-segmented contours as a baseline and to record the time. Quantitative metrics before and after using the auto-segmentation model were analyzed. The reduction in time was also analyzed. Participants were asked to complete a questionnaire to estimate the feasibility and efficacy of the auto-segmentation model and to comment on whether the auto-segmented contours were different from the routine contours of their institutions.

*Question 1. Are you satisfied with auto-segmentation for actual clinical use?*

*Question 2. Do you think that AI-based auto-contouring system will be able to replace human beings in the future?*

*Question 3. Do you think that that AI-based auto-contouring system will be helpful for physicians and/or dosimetrists in actual real-world clinic?*

Answer options: Not at all/No/Average/Yes/Definitely yes

## 3 Results

**Figure 2:**
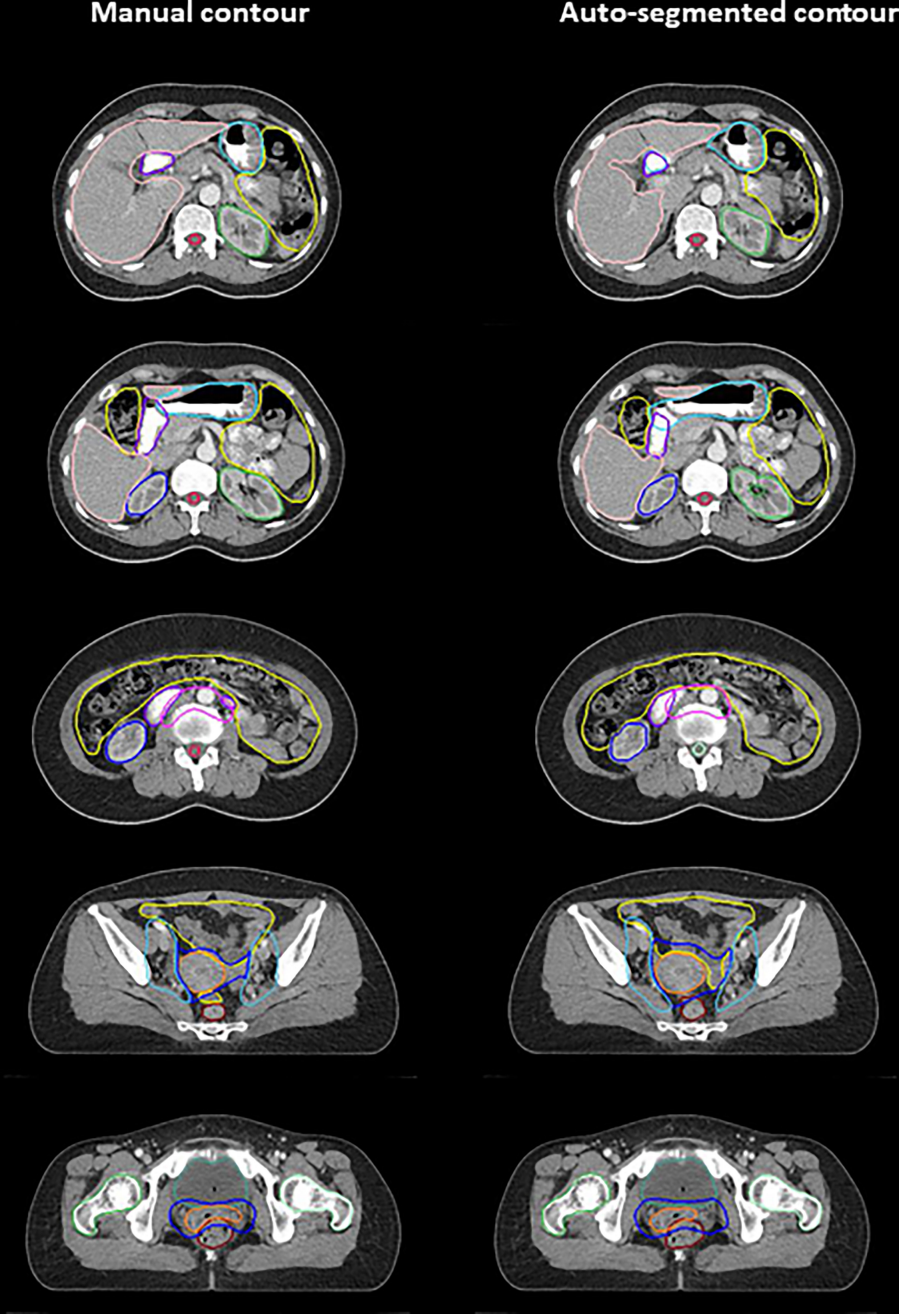
Example of deep learning-based auto-segmentation and manual contours.

Examples of deep-learning-based auto-segmented and manually contoured OARs/CTVs are shown in **Figure 2**. **Table 1** shows a comparison of the manual and auto-segmented contours obtained by DSC and HD. Regarding OARs, the anorectum, bladder, spinal cord, cauda equina, and right and left femoral heads showed DSCs between 0.80 and 0.90. Bowel bag, uterocervix, liver, left kidney, and right kidney showed excellent DSCs of over 0.90. For the stomach and duodenum, DSCs were 0.67 and 0.73, respectively. CTV1, CTV2, and CTV3 showed DSCs between 0.75 and 0.80. For HD, the anorectum, bladder, uterocervix, spinal cord, cauda equina, liver, right and left kidney, and right and left femoral head showed HD below 10 mm, while the bowel bag, stomach, duodenum, CTV1, CTV2, and CTV3 showed HD over 10 mm.

**Table 1:** Comparison of deep learning auto-segmentation and manual contours of organs-at-risk and clinical target volumes.

	DSC	SD	HD (mm)	SD (mm)
Organs-at-risk				
Stomach	0.67	0.27	28.33	29.28
Duodenum	0.73	0.19	12.60	13.50
Liver	0.94	0.01	6.20	1.47
Right kidney	0.90	0.03	7.60	3.50
Left kidney	0.90	0.04	8.20	4.83
Spinal cord	0.82	0.06	8.40	8.48
Cauda equina	0.81	0.04	5.20	3.69
Bowel bag	0.90	0.02	13.40	5.40
Bladder	0.88	0.24	6.93	13.41
Uterocervix	0.90	0.05	7.27	4.68
Anorectum	0.86	0.04	8.00	8.04
Right femoral head	0.89	0.07	7.47	4.79
Left femoral head	0.89	0.08	7.60	4.94
Target				
CTV1_primary	0.76	0.07	15.67	6.13
CTV2_pelvic node	0.77	0.05	12.53	5.73
CTV3_para-aortic node	0.80	0.12	13.00	8.69

DSC, Dice similarity coefficient; STD, standard deviation; HD, 95% Hausdorff distance; CTV, clinical target volume.

**Figure 3:**
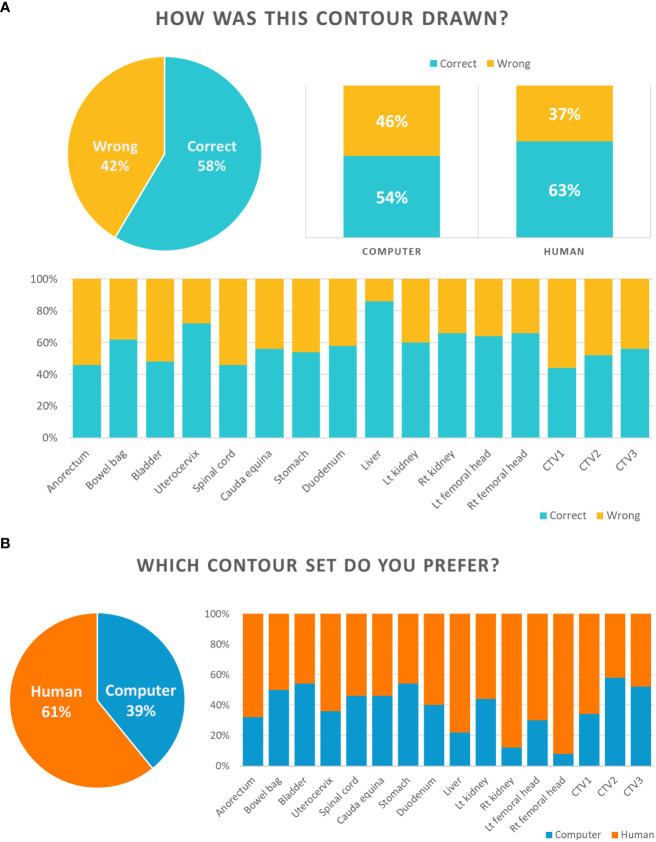
Results for Section I of the Turing test **(A)** and Section II of the Turing test **(B)**.

The results for Section I of the Turing test are shown in **Figure 3A**. For section I, a CT slice image of a single contour was provided, and participants were asked whether the contour was delineated by a human being or computer. Overall, the participants answered correctly for 58% of the questions, but expected differently for the other 42%. For auto-segmented and manual contours, the participants predicted correctly for 54% and 63% of the questions, respectively. Specifically, most participants correctly answered the question of whether the contour was drawn by a computer or a human in relation to the uterocervix and liver.

The results for Section II are shown in **Figure 3B**. The participants answered in favor of the auto-segmented contours and manual contours for 39% and 61% of the overall questions, respectively. The liver, right kidney and right and left femoral head were the OARs that the participants preferred manual contours over auto-segmented contours by more than 70%.

**Figure 4:**
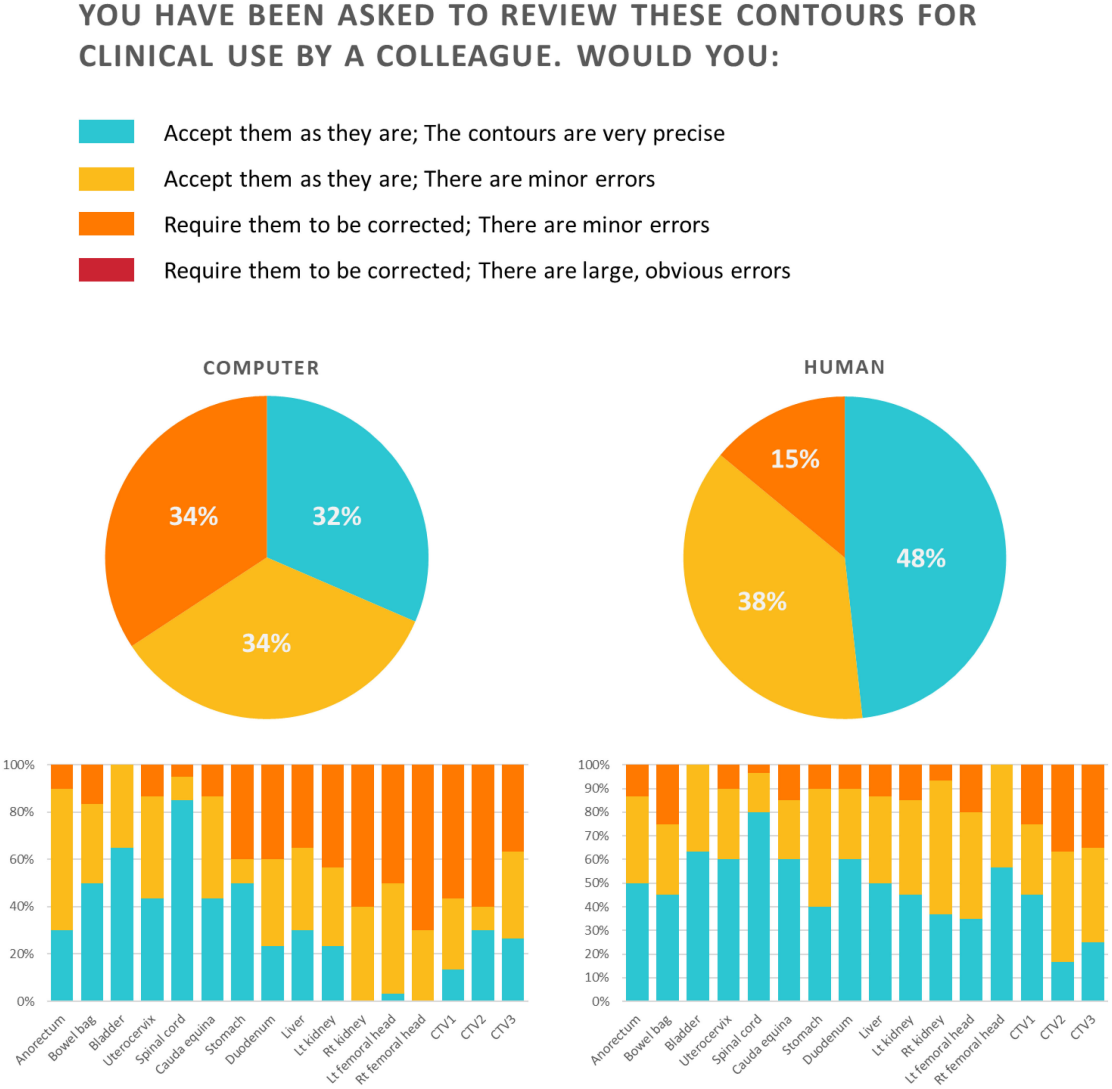
Results for Section III of the Turing test.

The results of Section III are shown in **Figure 4**. For the auto-segmented contours, which were blinded, the participants answered that they would accept them as they are for 66% and that they would require them to be corrected since they have minor errors for 34%. No auto-segmented contours were judged to have large, obvious errors. For the anorectum, bowel bag, bladder, uterocervix, spinal cord, and cauda equina, over 80% of the participants accepted the contours. In comparison, for the right kidney, left femoral head, right femoral head, CTV1, and CTV2, over 50% required correction for minor errors. For manual contours, the participants answered that they would accept them as they were for 86% and that they would require them to be corrected since they had minor errors for 14%. No large, obvious errors were observed for manual contours. More than 80% of the manual contours for all OARs, except for the bowel bag, were accepted as they were.

For section IV, participants were asked to provide an overall satisfaction score for five cases with auto-segmented contours (**Supplementary Figure 2**). The median overall satisfaction score was 7 (range 1–10). The median scores for each participating physician varied widely (range 3–9).

**Figure 5:**
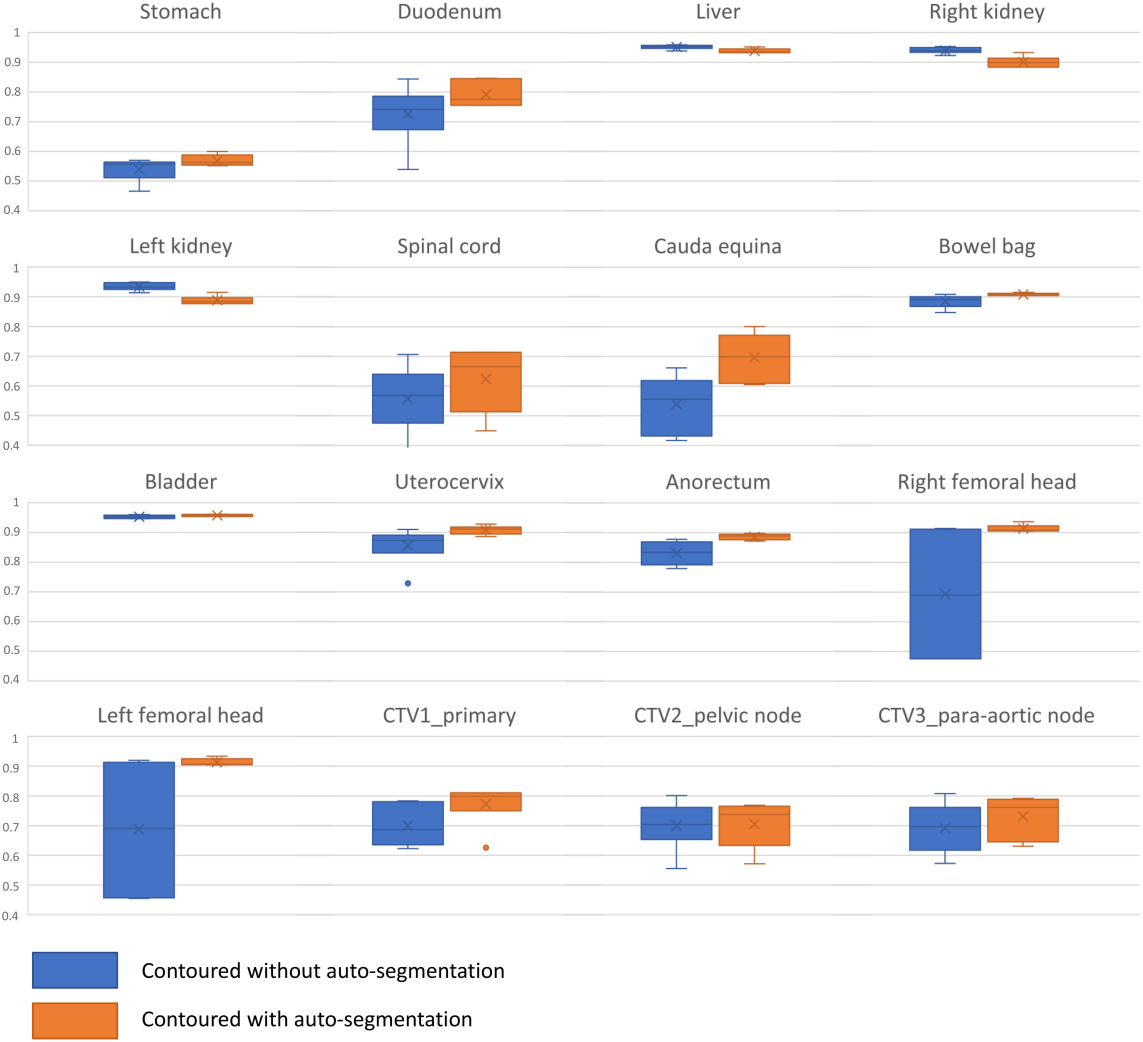
Boxplot for Dice similarity coefficient comparison between the original manual contour and contours delineated by the radiation oncologists in other institutions without and with auto-segmentation.

The comparison between the original manual contour and the OAR/CTVs contoured by radiation oncologists from other institutions with and without auto-segmentation is shown in **Table 2** and also as a boxplot in **Figure 5** for DCS and **Supplementary Figure 3** for HD. Among OAR/CTVs, the cauda equina, stomach, and spinal cord showed DSCs lower than 0.60 and the stomach and spinal cord showed HDs greater than 40 mm for contours without auto-segmentation. In contrast, the right and left kidneys, liver, and bladder showed DSCs higher than 0.90, and the right and left kidneys and bladder showed HDs smaller than 10 mm. For contours obtained using auto-segmentation, the stomach showed a DSC lower than 0.60, and the stomach and spinal cord showed HDs greater than 40 mm. The right and left kidney, bowel bag, uterocervix, right and left femoral head, liver, and bladder showed DSCs higher than 0.90, and the right and left femoral head and bladder showed HDs smaller than 10 mm. Overall, the mean DSC and HD for the contours without using auto-segmentation were 0.76 (standard deviation [SD] 0.07) and 25.11 (SD 16.51), respectively. For contours using auto-segmentation, the overall mean DSC and HD were 0.82 (SD 0.03) and 24.40 (SD 7.34), respectively, showing slightly a higher DSC and a smaller HD with smaller SDs suggesting reduced heterogeneity. The median times for contouring all OAR/CTVs without auto-segmentation and with auto-segmentation were 82 min (range, 66–101 min) and 51 min (range, 31–78 min), respectively. The responses to Questions 1, 2, and 3 are shown in **Supplementary Figure 4**. Most participating radiation oncologists scored average or in favor of the auto-contouring system. All participants, except one, answered that the CTVs were different from those of their institution, but that the OARs were similar. Most comments concerning the CTVs were about the margin and the range of muscle or vessel the CTV included.

**Table 2:** Comparison between the original manual contour and contours delineated by the radiation oncologists in other institutions without and with auto-segmentation.

	Manual				Auto-segmentation-based			
	DSCMean	SD	HDMean	SD	DSCMean	SD	HDMean	SD
Stomach	0.54	0.04	54.70	24.54	0.57	0.02	64.50	4.41
Duodenum	0.73	0.09	24.02	14.30	0.79	0.04	18.83	1.14
Liver	0.95	0.01	11.67	7.38	0.94	0.01	18.57	0.85
Right kidney	0.94	0.01	9.49	6.01	0.90	0.02	19.39	1.42
Left kidney	0.93	0.01	7.96	4.75	0.89	0.01	17.60	1.22
Spinal cord	0.56	0.10	49.15	59.16	0.62	0.10	56.48	54.95
Cauda equina	0.54	0.09	31.26	14.60	0.70	0.08	24.57	9.93
Bowel bag	0.89	0.02	27.39	19.15	0.91	0.00	33.33	9.64
Bladder	0.95	0.01	5.21	2.22	0.96	0.00	5.51	0.37
Uterocervix	0.86	0.06	18.43	8.87	0.91	0.01	18.25	2.41
Anorectum	0.83	0.04	13.07	9.58	0.89	0.01	10.44	3.30
Right femur head	0.69	0.22	33.04	25.96	0.91	0.01	8.63	1.63
Left femur head	0.69	0.22	33.59	26.45	0.91	0.01	8.57	1.94
CTV1_primary	0.70	0.06	36.48	17.38	0.77	0.07	36.82	9.92
CTV2_pelvic node	0.70	0.08	22.93	12.37	0.71	0.07	21.91	6.26
CTV3_para-aortic node	0.69	0.08	23.40	11.48	0.73	0.06	27.03	7.98
***Mean***	***0.76***	***0.07***	***25.11***	***16.51***	***0.82***	***0.03***	***24.40***	***7.34***

DSC, Dice similarity coefficient; SD, standard deviation; HD, 95% Hausdorff distance; CTV, clinical target volume.

## 4 Discussion

We trained a deep learning-based auto-segmentation model for 13 OARs and three CTVs using simulated CT images of patients with cervical cancer. The proposed auto-segmentation model showed acceptable agreement with geometric indices, such as DSC and HD, and favorable clinical agreement with various subjective evaluation methods.

Unlike studies applying artificial intelligence in other medical areas, one specific characteristic of studies reporting auto-segmentation is that the concept of ground truth is disputable. The contours of OAR/CTVs vary among physicians, even within the same institution (5). Therefore, although the current model was generated with contours according to existing guidelines, the contours of the current model may not fit all clinical situations. Multi-domain evaluation may be necessary to evaluate auto-segmentation for RT, because geometric indices such as DSC and HD are not well correlated with clinically meaningful endpoints (22). We included geometric indices for evaluation and qualitative evaluation methods, such as the Turing test, assessing changes in inter-physician variability and contouring time, and physician-reported assessment.

Previously, studies have reported the results of auto-segmentation of OAR/CTVs in cervical cancer (15, 23–26). One study including 100 cases for model training reported similar results to those of our study for OARs (24). The DSC for the CTV in this study was slightly higher, but in our study, CTVs were divided into CTV1, CTV2, and CTV3, which may have affected the results. Small differences may translate into larger differences in DSCs in smaller contour volumes. In another study by Rhee et al., overall, there were no large differences compared to the results of this study (15). The differences in DSC values for primary CTV and spinal cord may be attributed to the higher number of CT scans (2254 cases) included in the model by Rhee et al. (15). A study by Li et al. employed quantitative metrics, including the DSC, HD, and true positive volume fraction, to analyze the contours of the CTV, bladder, rectum, bowel bag, and femoral head in postoperative cases. The DSC values ranged from 0.84 to 0.93 (25). In another study, in addition to DSC and HD, the Jaccard coefficient and dose-volume index were utilized for dosimetric evaluation of the spinal cord, kidney, bladder, femoral head, pelvic bone, rectum, and small intestine (26). Recent studies have suggested that surface metrics, such as surface DSC and Added Path Length, are better tools for analyzing clinical acceptability or correlating with time-savings compared to traditional metrics such as DSC and HD (27–30). However, calculating surface DSC and Added Path Length is not yet possible using commercial software and thus cannot be performed in general. In our study, we used traditional geometric indices such as DSC and HD, which may be a limitation, but we also incorporated various subjective evaluation methods to overcome this limitation.

Currently, the progress in this field has been exponential and there are several commercial deep-learning based auto-segmentation software for the female pelvis such as Therapanacea Annotate, AccuContour, AI-Rad Companion, OncoStudio et cetera. Therapanacea Annotate provides 14 OARs and 10 LN contours (Anal Canal, Bladder, Bowel Bag, External Contour, Femoral Heads, Iliac, Kidneys, Liver, Medullar Canal, Rectum, Sigmoid, Spinal Cord, Common Iliac Gyneco LN, CTVt Gyneco, Iliac Gyneco LN, Inguinal Gyneco LN, Lomboaortic Gyneco LN, Parametrium, Presacral Gyneco LN, Vagina) and AccuContour and OncoStudio currently provides OAR contours such as Bowel bag, Small intestine, Rectum, Bladder, Femoral head, Liver, Kidney, Stomach, Duodenum. One of the strengths of the current model in this study would be the inclusion of CTVs compared to the products that only include OARs.

Our study has several strengths; in that, it included numerous OAR/CTVs, which were more diverse than previous studies. In the current study, stomach and duodenum showed poorer DSCs compared to the other OARs. The superior limit of CT images, which may include all or part of the stomach, may have affected the results. The duodenum is a small organ that is difficult to contour manually; thus, a lower DSC compared to other OARs can be expected.

The Turing test showed that the auto-segmented OAR/CTVs were comparable to the manual contours. No auto-segmented contours were considered to have large, obvious errors, although the percentage of contours requiring correction for minor errors was higher than that for manual contours. Participants preferred manual contours over auto-segmented contours for the following OARs: liver, right kidney, and right and left femoral head. This may be due to the fact that the auto-segmentation model contours the OARs too accurately, as shown in an example of a liver contour in **Supplementary Figure 5**. This is an interesting point in the evaluation of auto-segmentation models, showing that highly “accurate” contours do not directly translate into contours that are favored in real-world clinical practice. Overall, most radiation oncologists completing the Turing test were satisfied with the auto-segmentation model.

When radiation oncologists from six different institutions were asked to contour all OAR/CTVs on an anonymized CT scan with or without auto-segmentation, reductions in contouring time were observed. We also aimed to analyze inter-physician heterogeneity, but statistical tests could not be applied due to the small sample size. The cauda equina, stomach, and spinal cord showed the lowest DSC when the contours drawn by the participating physicians were compared to the manual contours originally used for model training. The cauda equina and spinal cord are simple OARs to contour, but relatively minor differences, such as contouring the spinal cord with a circumference of 7 mm or 8 mm or setting the superior or inferior margin differently, may have largely affected the geometric indices. In addition, setting the superior margin differently and contouring the stomach on all available CT slices or only on clinically relevant CT slices considering the treatment field may have affected the results. Interestingly, the contours for both kidney and liver tended to show lower DSC after using auto-segmentation compared to those without auto-segmentation. One possible explanation for this may be the over-accurately contoured OARs by auto-segmentation, as previously mentioned. Some of the participants may have viewed the auto-segmented OARs as clinically acceptable and may have not modified the contours, which could have affected the DSC which is calculated using the original manual contours for comparison.

Using the auto-segmentation system reduced the mean contouring time by approximately 30 min and 84% of the physicians were satisfied with the auto-segmentation model considering auto-segmentation to be helpful in clinical practice. Furthermore, 84% of physicians considered that the deep learning-based auto-segmentation system would be able to replace humans in the future, despite the limitations of the current model. Almost all participants stated that contouring was different from their institution’s routine practice. The guidelines contain limited detail; hence, routine practice for the target volumes may vary by institution and physicians. This poses a task and a limitation for AI-based auto-segmentation.

However, it seems that AI-based auto-segmentation can be effectively used in multicenter clinical trials, because RT quality assurance, including consistent contouring and planning in clinical trials, is known to affect the results (6, 31). As contouring is the first step of treatment, it may be useful to use auto-segmentation to minimize deviations between institutions.

One limitation of this study was the number of CT images included to train the model. However, the geometric indices were similar to those in other studies, and the contours were clinically accepted without any major errors. Second, only contrast-enhanced CT images were used for the training; hence, the role of this auto-segmentation model may be limited with non-contrast CT scans. In addition, one radiation oncologist contoured all manual contours. This ensured the availability of homogeneous data for deep learning. However, variations in contours exist in the real world, and using only homogenous data may limit the generalization of the model. Finally, the small number of validation set is also a limitation considering the application for general use. Further external validation using data from other institutions is needed and will be planned as an additional project in the future.

## 5 Conclusion

This study demonstrated the potential effectiveness and feasibility of a deep learning-based auto-segmentation model for patients with cervical cancer undergoing RT. Although there may be limitations of the current model as a complete substitute for human beings, it can serve as a useful and effective tool in real-world clinical practice.

## Data availability statement

The original contributions presented in the study are included in the article/**Supplementary Material**. Further inquiries can be directed to the corresponding author.

## Ethics statement

The studies involving human participants were reviewed and approved by Institutional Review Board of Severance Hospital. Written informed consent for participation was not required for this study in accordance with the national legislation and the institutional requirements.

## Author contributions

SC, JC, and YK contributed to conception and design of the study. SC organized the database and performed the statistical analysis. SC and JC wrote the first draft of the manuscript. SC, JC, and YK wrote sections of the manuscript. All authors contributed to the article and approved the submitted version.
