# Supplementary material for: Comprehensive clinical evaluation of deep learning-based auto-segmentation for radiotherapy in patients with cervical cancer
Source: Front Oncol. 2023 Apr 28;13:1119008. doi: 10.3389/fonc.2023.1119008 (PMC10175826; doi:10.3389/fonc.2023.1119008)
Supplement: Supplementary file 1 [file DataSheet_1.docx]

Supplementary Material

# Supplementary Data

## Supplementary Material

*Explanation of the deep learning algorithm*

The first stage identified the approximate size and location of the OAR/CTV to be divided and the second stage was used to perform contouring within the volume-of-interest (VOI) of each OAR/CTV. The second stage was not performed for OARs that were small or had minimum shape variation, such as the spinal cord, cauda equina, and femoral head. For the inputs of the convolutional neural network (CNN), the image intensity values of a truncated range of [-160, 240] were linearly normalized to the range of [0, 1]. The data for the first stage were resized to a resolution of 1 mm × 1 mm × 3 mm, and a patch was created by moving in 64 × 64 × 10 voxels with a size of 160 × 160 × 32 voxels. The VOI was obtained using the prediction result of the first stage and used as an input for the second stage. The bounding box of each OAR was set as the VOI, and a margin of as many as 32 × 32 × 10 voxels was given to obtain one patch. The obtained patch was resized to a network input size of 160 × 160 × 32 voxels. In the case of the CTV, the bounding box of the bowel bag and anorectum combined among the first-stage prediction results was set as the VOI. The input size of the model for the CTV segmentation was 96 × 96 × 128 voxels. A U-Net structure using EfficientNet-B0 as the backbone was used as the network model. The same network structure was used for both the first and second stage, and only the last layer was changed to match the number of target organs in each stage. A combination of binary cross-entropy and Dice loss was used for the loss function.

## Supplementary Figures


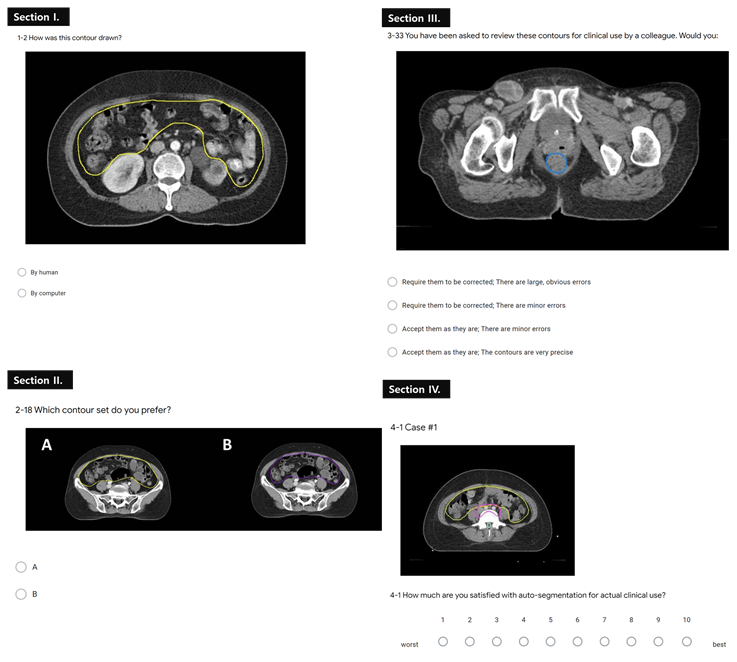


**Supplementary Figure 1.** Example of the Turing test.


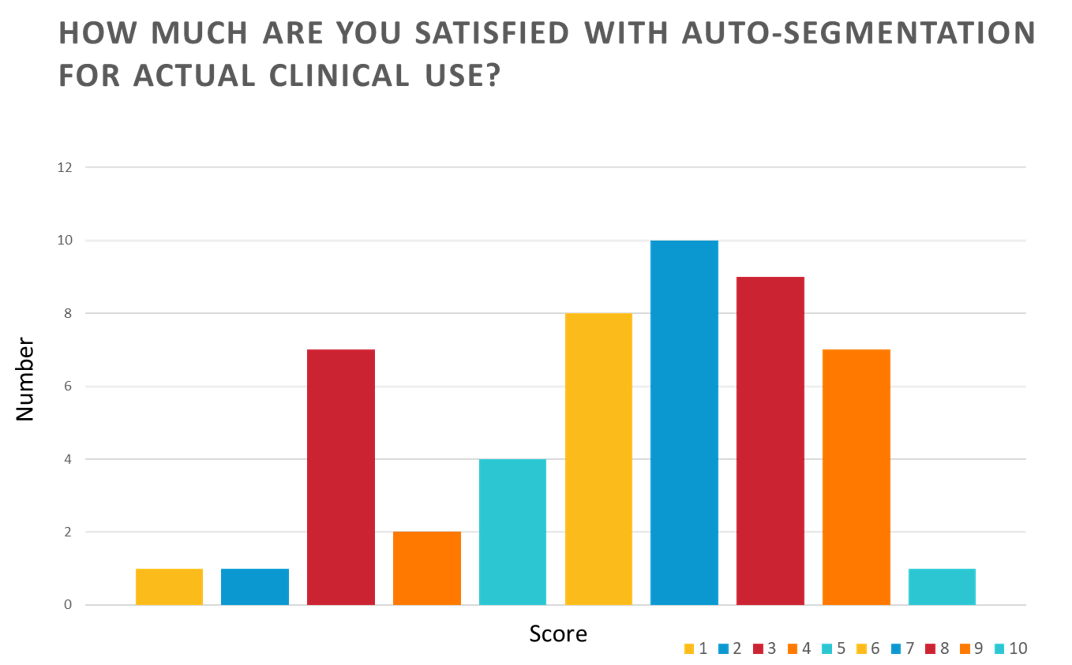


**Supplementary Figure 2.** Results for Section IV of the Turing test.


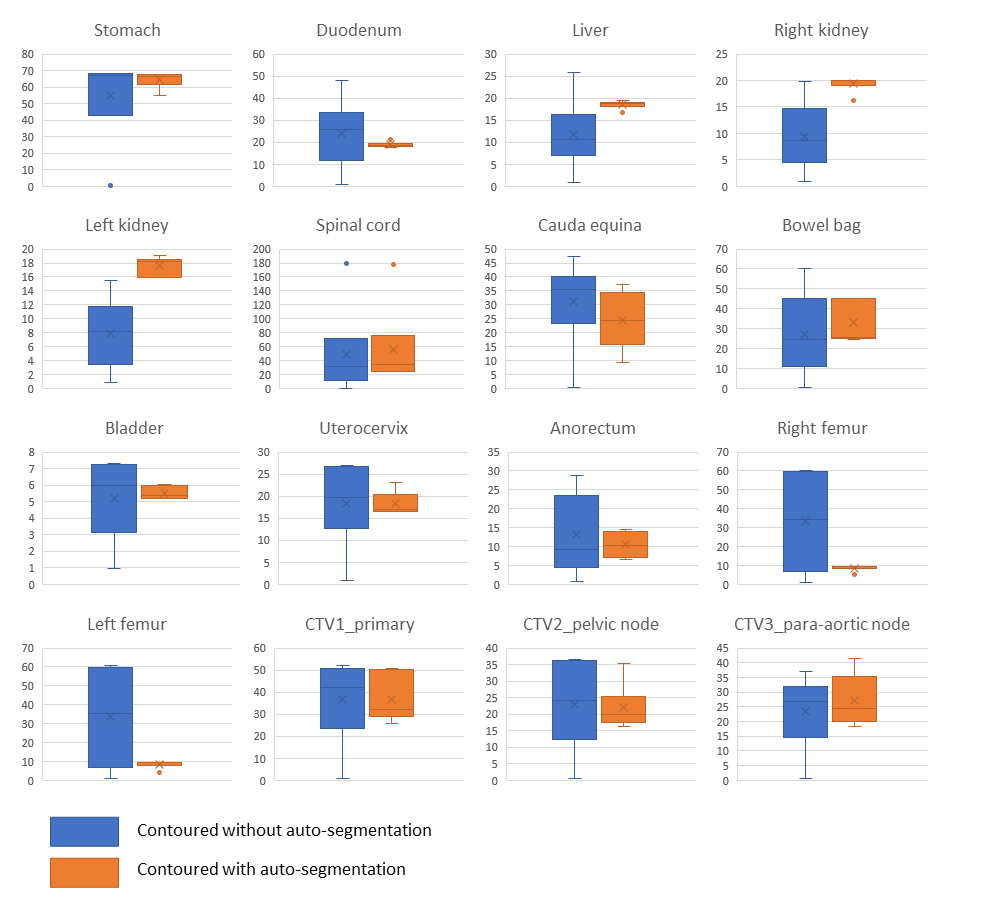


**Supplementary Figure 3.** Boxplot for Hausdorff distance comparison between the original manual contour and contours delineated by the radiation oncologists in other institutions without and with auto-segmentation.


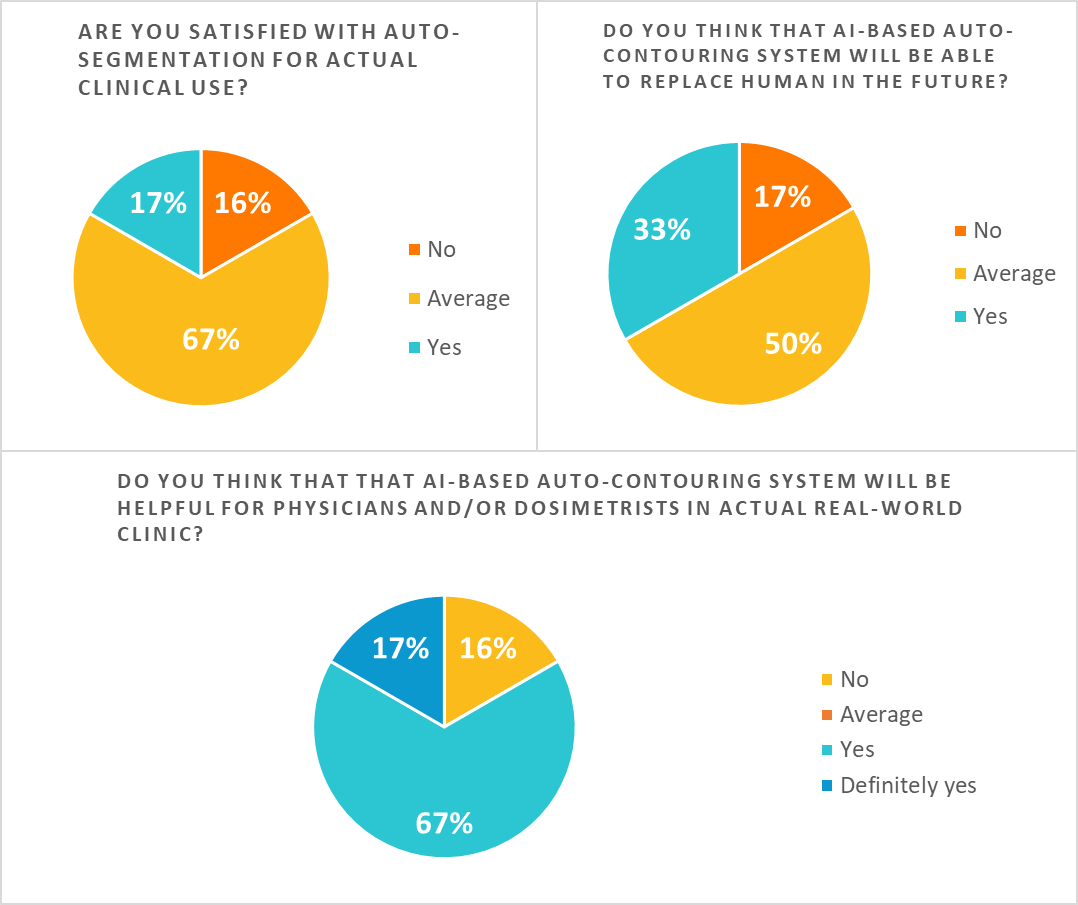


**Supplementary Figure 4.** Responses to the questionnaire.


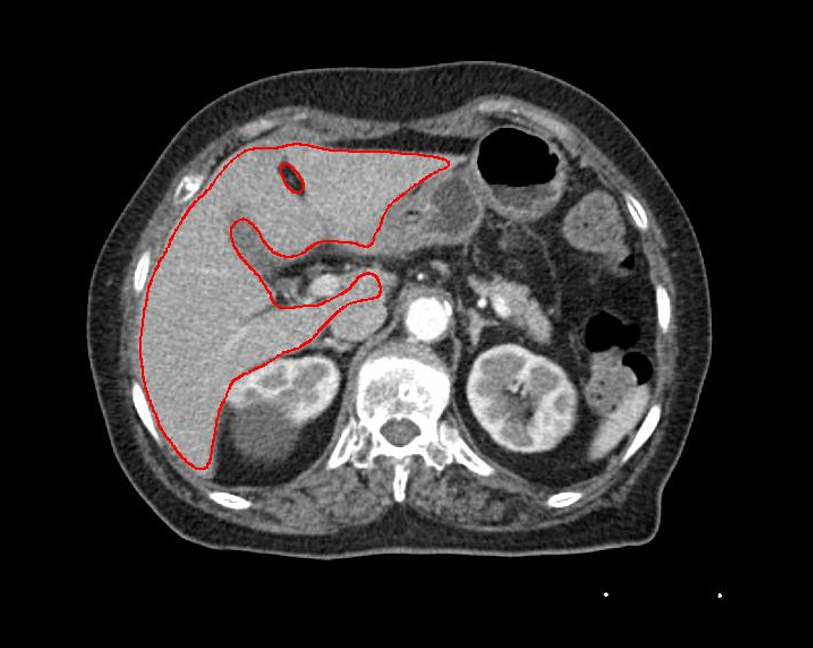


**Supplementary Figure 5.** Example of over-accurately delineated liver contour.
